# Key Parameters Requirements for Non‐Fullerene‐Based Organic Solar Cells with Power Conversion Efficiency >20%

**DOI:** 10.1002/advs.201802028

**Published:** 2019-03-10

**Authors:** Yuliar Firdaus, Vincent M. Le Corre, Jafar I. Khan, Zhipeng Kan, Frédéric Laquai, Pierre M. Beaujuge, Thomas D. Anthopoulos

**Affiliations:** ^1^ King Abdullah University of Science and Technology (KAUST) KAUST Solar Center (KSC) Division of Physical Sciences and Engineering Thuwal 23955–6900 Saudi Arabia; ^2^ University of Groningen Zernike Institute for Advanced Materials Nijenborgh 4 9747 AG Groningen The Netherlands; ^3^ Department of Physics Imperial College London South Kensington London SW7 2AZ UK

**Keywords:** bulk‐heterojunction solar cells, drift‐diffusion model, nonfullerene acceptors, numerical device simulations, organic photovoltaics, tandem devices

## Abstract

The reported power conversion efficiencies (PCEs) of nonfullerene acceptor (NFA) based organic photovoltaics (OPVs) now exceed 14% and 17% for single‐junction and two‐terminal tandem cells, respectively. However, increasing the PCE further requires an improved understanding of the factors limiting the device efficiency. Here, the efficiency limits of single‐junction and two‐terminal tandem NFA‐based OPV cells are examined with the aid of a numerical device simulator that takes into account the optical properties of the active material(s), charge recombination effects, and the hole and electron mobilities in the active layer of the device. The simulations reveal that single‐junction NFA OPVs can potentially reach PCE values in excess of 18% with mobility values readily achievable in existing material systems. Furthermore, it is found that balanced electron and hole mobilities of >10^−3^ cm^2^ V^−1^ s^−1^ in combination with low nongeminate recombination rate constants of 10^−12^ cm^3^ s^−1^ could lead to PCE values in excess of 20% and 25% for single‐junction and two‐terminal tandem OPV cells, respectively. This analysis provides the first tangible description of the practical performance targets and useful design rules for single‐junction and tandem OPVs based on NFA materials, emphasizing the need for developing new material systems that combine these desired characteristics.

## 1 Introduction

Organic nonfullerene acceptor (NFA) molecules have nowadays successfully replaced their fullerene counterparts in organic photovoltaics (OPVs) while offering the potential for large‐scale industrial production and commercialization.^1^ In the past few years rapid progress has been made in the development of a variety of NFAs, including both polymeric and small molecule organics. These NFAs have been paired with donor materials in a variety of OPV device platforms including single‐junction and multijunction solar cells.^2^, ^3^, ^4^, ^5^, ^6^, ^7^ The best power conversion efficiencies (PCEs) of OPVs have exceeded 14% in single‐junction devices^2^, ^4^ and over 17% in two‐terminal (2T) tandem devices.^3^ Compared to fullerene acceptor–based devices, they exhibit not only higher PCE but also improved morphological and photochemical stability,^2^, ^4^, ^8^, ^9^, ^10^, ^11^, ^12^ while offering significant promise for further performance improvement.^13^ To enable further performance increases, the factors limiting the OPV efficiency must be identified. Detailed insights into the avoidable losses will help direct researchers to design and develop new material systems with desired characteristics.

Previous studies have predicted that the NFA‐based OPVs can exceed PCE of 20% for single‐junction and 25% for the 2T monolithic tandem devices.^3^, ^13^, ^14^, ^15^ While those studies have used simple empirical models, we use a commercially available 1D numerical drift‐diffusion device simulator to re‐evaluate the efficiency limit for NFA‐based OPVs. The charge extraction of various type of OPVs have been successfully explained on the basis of drift‐diffusion simulations which assumed time‐independent bulk mobilities (e.g., from space‐charge‐limited current (SCLC) technique).^16^, ^17^, ^18^ The simulation numerically solves the drift‐diffusion equations that govern the current–voltage (*J–V*) characteristics of the device, thus taking into account charge generation, recombination effects, and the carrier mobilities of the active layer. Koster et al. used a numerical drift‐diffusion model to simulate polymer:fullerene OPVs for which an efficiency of 10.8% was predicted for electron mobility of 2 × 10^−3^ cm^2^ V^−1^ s^−1^.^19^ However, a high lowest unoccupied molecular orbital (LUMO)–LUMO offset of 0.5 eV was assumed and the effect of charge mobility and recombination rate constant were not explicitly treated. To this end, Würfel et al. performed drift‐diffusion simulations and explicitly included charge carrier mobility in their estimates of achievable efficiencies, predicting a maximum PCE of ≈22% for material systems with carrier mobility values of >10^−2^ cm^2^ V^−1^ s^−1^, and a LUMO–LUMO offset of 0.2 eV.^20^ In the latter work, however, the workers assumed complete absorption of photons in the AM1.5G spectrum with energies larger than the bandgap of the donor material. With the rapidly advancing field of NFA materials and resulting OPVs, it is imperative that the modeling approaches and relevant predictions have to be adjusted accordingly. Here, we assume realistic absorption profiles for the donor and NFA acceptor materials with varying bandgaps. Using a drift‐diffusion model we are able to treat explicitly the effect of several different parameters including, active‐layer absorption, layer thicknesses, charge carrier mobility and recombination rate constant. This holistic approach allow us to determine to what extent the mobility of new materials should be increased and the level at which the recombination rate constant should be reduced in order to reach the performance targets of next generation NFA‐based OPVs.

To this end, we explicitly show that NFA‐based OPV cells with active layer thickness down to 100 nm could reach PCE values in excess of 18%, if the active layer can be designed to combine broad optical absorption (achievable by combining a donor and an acceptor with bandgaps of 1.7 and 2 eV, respectively) with a balanced hole and electron mobility value of 5 × 10^−4^ cm^2^ V^−1^ s^−1^, and a nongeminate recombination rate constant (*k*) of 1 × 10^−12^ cm^3^ s^−1^. Moreover, we highlight the importance of maintaining the balance between photocurrent gain—resulting from broad absorption characteristics of the active layer—and open circuit voltage (*V*_OC_) reduction due to the incorporation of low bandgap materials. Further improving the hole and electron mobilities of the active layer to >10^−3^ cm^2^ V^−1^ s^−1^ and the active layer thickness to >200 nm would raise single‐junction *PCE* values to over 20%. We also find that reducing *k* by one order of magnitude, from 10^−12^ to 10^−13^ cm^3^ s^−1^, translates to a similarly high PCE value of 20%. Further examination of the PCE limit of 2T tandem NFA OPVs yields predicted PCEs values of up to 25%, as long as the hole and electron mobilities in both subcells remain balanced and are on the order of 10^−3^ cm^2^ V^−1^ s^−1^, while the recombination rate constant is reduced to 10^−12^ cm^3^ s^−1^. Our simulations indicate that the performance of single‐junction and tandem OPV device efficiency will continue to improve as higher carrier mobility materials with matching optical bandgaps are being developed, while highlighting the specific material and device characteristics required for achieving optimum cell performance.

## 2 Results and Discussion

**Figure 1:**
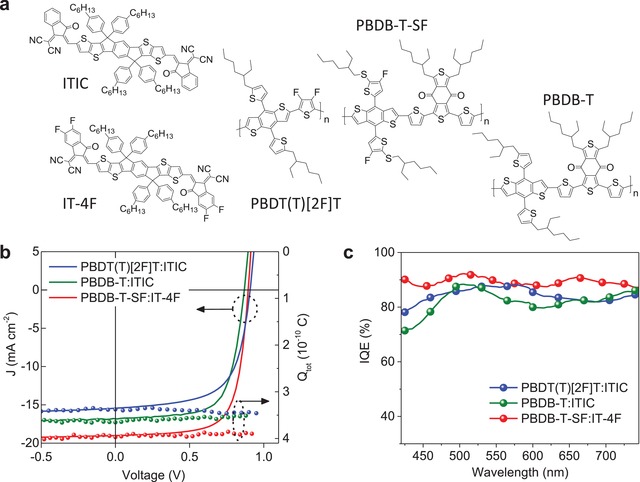
a) Molecular structure of donor (D) and acceptor (A) materials used in this study. b) *J–V* characteristics and total amount of charge (*Q*_tot_) extracted from the device as a function of pre‐bias (*V*_pre_) measured by time delayed collection field (TDCF) experiments (10 ns delay time, −4 V collection bias, laser pulse fluence: 0.1 µJ cm^−2^). c) IQE spectra of PBDT(T)[2F]T:ITIC, PBDB‐T:ITIC, and PBDB‐T‐SF:IT‐4F BHJ cells.

In order to validate the device simulator, several high‐efficiency NFA‐based OPV devices were fabricated, including: PBDT(T)[2F]T:ITIC, PBDB‐T:ITIC, and PBDB‐T‐SF:IT‐4F (**Figure** **1**a).^8^, ^21^, ^22^ Figure 1b displays the experimentally measured current density‐voltage (*J–V*) characteristics while **Table** **1** summarizes the key device parameters including *V*_OC_, short‐circuit current density (*J*_SC_), fill‐factor (*FF*), PCE, and hole/electron mobilities (μ_h_/μ_e_). The PBDT(T)[2F]T:ITIC device yields the lowest performance with 62% *FF* and 9.1% PCE as it exhibits the lowest hole and electron mobility (≈10^−5^ cm^3^ V^−1^ s^−1^). The device performance increases gradually as the balanced carrier mobility increases and the PBDB‐T‐SF:IT‐4F device was optimized with 12% PCE and 71% *FF*. Additionally, due to its low mobility, the optimized condition for the PBDT(T)[2F]T:ITIC device was achieved with an active layer thickness of ≈70 nm. On the other hand, the higher of mobility of PBDB‐T:ITIC and PBDB‐T‐SF:IT‐4F devices allows use of a thicker layer (≈100 nm).

**Table 1:** Average hole (μ_h_) and electron (μ_e_) mobility and photovoltaic performance of PBDT(T)[2F]T:ITIC, PBDB‐T:ITIC, and PBDB‐T‐SF:IT‐4F NFA‐based OPV cells. Maximum theoretical *J*_SC_ (*J*_SC,max_) was calculated via transfer matrix model by assuming 100% IQE. Charge‐carrier mobility of PBDB‐T and PBDB‐T‐SF‐based BHJ devices were inferred from space‐charge‐limited current (SCLC) (see the Supporting Information) while for PBDT(T)[2F]T‐based device were obtained from metal‐insulator CELIV (mis‐CELIV) measurements^21^

BHJ system	Thickness [nm]	μ_h_ [cm^2^ V^−1^ s^−1^]	μ_e_ [cm^2^ V^−1^ s^−1^]	*V*_OC_ [V]	*J*_SC_ [mA cm^−2^]	*J*_SC,max_ [mA cm^−2^]	*FF* [%]	PCE [%]
PBDT(T)[2F]T:ITIC	70	3.0 × 10^−5^	1.2 × 10^−5^	0.940	15.7	19.9	62	9.1
PBDB‐T:ITIC	100	3.3 × 10^−4^	1.0 × 10^−4^	0.870	16.8	20.2	68	10.0
PBDB‐T‐SF:IT‐4F	100	1.2 × 10^−4^	2.5 × 10^−4^	0.895	19.4	21.7	67	11.7

The *FF* difference in these representative devices may also be caused by field‐dependent charge generation in compe‐tition with nongeminate recombination. To either confirm or refute this hypothesis we conducted time‐delayed collection field (TDCF) measurements.^23^, ^24^ Figure 1b shows the TDCF results for the representative NFA‐based OPV cells following optical excitation at 532 nm for delay times of 10 ns, and across a range of prebias conditions between −1 to *V*_OC_ (0.9 V). Interestingly, the generation of charges (*Q*_tot_) in all devices is found to be independent of the applied prebias. This finding suggests that geminate pair splitting efficiency is electric‐field independent in line with recent findings for the state‐of‐the‐art bulk‐heterojunction (BHJ) OPVs.^24^, ^25^, ^26^, ^27^, ^28^, ^29^, ^30^ Here, however, the *FF* of the NFA‐based OPVs is found not to be influenced by the geminate recombination, but instead dictated by nongeminate recombination processes. The internal quantum efficiency (IQE) spectra of the cells are displayed in Figure 1c, calculated by disregarding its dependence on optical absorption. Noticeably, IQE of PBDB‐T‐SF:IT‐4F cells is around 90% while average IQE for BHJ devices based on PBDB‐T and PBDT(T)[2F]T are between 82–84%. The aforementioned IQEs are in agreement with those estimated from the measured *J*_SC_ divided by the *J*_SC,max_ calculated by transfer matrix simulations (Table 1).

**Figure 2:**
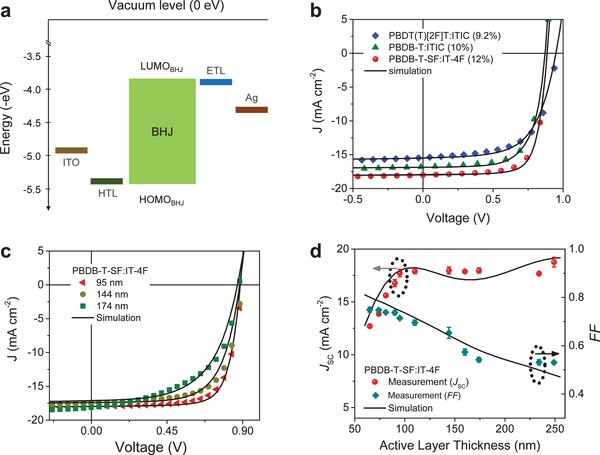
a) Schematic energy diagram for single‐junction BHJ OPV cell with normal device structure. b) Experimentally measured (symbol) and simulated (solid line) current‐density–voltage (*J–V*) characteristics for PBDT(T)[2F]T:ITIC, PBDB‐T:ITIC, and PBDB‐T‐SF:IT‐4F BHJ solar cells, c) and for PBDB‐T‐SF:IT‐4F BHJ solar cells of different thicknesses (95, 144, and 174 nm). d) Experimentally measured (symbol) and simulated (solid line) fill‐factor (*FF*) and short‐circuit current density (*J*_SC_) versus active layer thickness for PBDB‐T‐SF:IT‐4F BHJ solar cells. Input parameters used for the simulation can be found in Table S1 (Supporting Information).

To predict the NFA‐based OPV cell performance, we used the Setfos 4.4 commercial device simulator (FLUXiM AG). The numerical simulator utilizes transfer matrix modelling to determine the layer specific charge generation profile and numerically solves the drift‐diffusion equations, which include charge recombination (nongeminate) and extraction effects to simulate the *J–V* curve.^16^ The active layer of the BHJ solar cell is modelled using an effective medium approximation that considers the BHJ as a single‐phase material (the real interfaces between donor and acceptor are not explicitly considered). The highest occupied molecular orbital (HOMO) of the effective semiconductor BHJ (HOMO_BHJ_) is taken as the HOMO value of the polymer donor, while the LUMO of the effective semiconductor BHJ layer (LUMO_BHJ_) is taken as the LUMO value of the NFA (**Figure** **2**a). For our simulations, we used a device structure that closely matches our experimental devices (see the Experimental Section). Figure 2a shows the energy diagram of the electrodes, active‐layer, electron‐transport layer (ETL), and hole‐transport layer (HTL) for an OPV cell with normal structure. The work‐functions of the interlayers/electrodes are aligned with the corresponding energy levels of the BHJ layer (Figure 2a), forming Ohmic contacts (i.e., in this configuration, neither the HTL nor the ETL act as electron‐, hole‐blocking layers which we call metallic contact).

Following previous theoretical and experimental studies, several assumptions are used in this work: 1) only nongeminate recombination is included in our model as it is commonly considered to be the main recombination pathway limiting the performance of high‐efficiency OPVs, in excellent agreement with our TDCF measurements (Figure 1b).^27^, ^31^, ^32^ This assumption is justified as we focus solely on the simulation of high‐efficiency OPVs. The recombination rate *R* is described by the law of mass action (*R* = *k∙n∙p*), where *k* is the nongeminate recombination rate constant, and *n* and *p* are the electron and hole densities, respectively. 2) Here *k* is considered independent of charge carrier mobility parameters for reasons detailed in previously reported theoretical and experimental studies.^16^, ^33^, ^34^, ^35^ 3) The electron and hole mobilities were assumed to be electric‐field independent so that the number of fit parameters in the model is minimized.

The simulator is able to reconstruct the operating characteristics of fullerene‐based BHJ OPVs for a wide range of active layer thicknesses and charge carrier mobilities.^16^ Thus, we first simulated the operating characteristics of high‐efficiency PBDT(T)[2F]T, PBDB‐T, and PBDB‐T‐SF ‐based BHJ solar cells. For the purpose of *J–V* simulation, we first measured the optical constants, namely, refractive index and extinction coefficient, of all active layers using ellipsometry (Figure S3, Supporting Information). With the nongeminate recombination rate *k* as a fitting parameter, we were able to simulate the experimental device characteristics. Rate constant values of ≈1 × 10^−12^ cm^3^ s^−1^ for PBDT(T)[2F]T‐based cells, and ≈7 × 10^−12^ cm^3^ s^−1^ for PBDB‐T‐based device, were found to yield best fits between experiment and simulation. We note that the rate constant inferred from TDCF measurements (*k*_TDCF_) is higher than the one obtained from the simulations (Table S1, Supporting Information). It appears that the *k*_TDCF_ value represents more of an upper limit for the recombination as has been discussed in earlier work.^36^

Figure 2b shows that the simulated *J–V* curves (solid lines) closely resemble the experimentally measured *J–V* data (symbols). The device simulator also accurately reconstructs the *J–V* curves of PBDB‐T‐SF:IT‐4F BHJ solar cells of different active layer thicknesses (Figure 2c, and Figure S4 (Supporting Information) for PBDT(T)[2F]T‐based OPVs). For the latter device, the *FF* is varying from 70% to 50% and the short‐circuit current density (*J*_SC_) from 12 to 19 mA cm^−2^ when the active layer thickness is increased from 60 to 250 nm (Figure 2d), respectively. The excellent agreement between the experimental and simulated *J–V* curves for different material systems and layer thicknesses, demonstrates that the simulator tool is able to capture the physical processes that govern charge carrier photogeneration, transport and recombination in these NFA‐based BHJ OPVs.^16^

**Figure 3:**
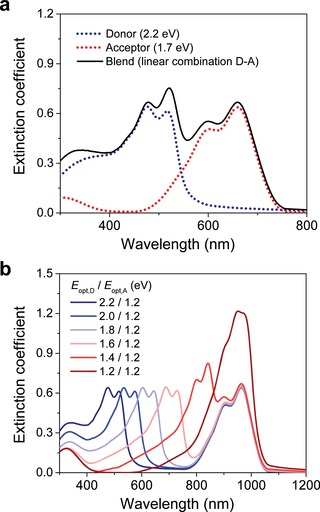
a) Example of how the extinction coefficient spectra of BHJ active layer considered in this study was produced from a linear combination of the extinction coefficient of donor (PBDT(T)[2F]T) and acceptor (ITIC). b) Generated extinction coefficient spectra of several BHJ films used for the simulation and obtained by shifting the spectra of donor from 2.2 to 1.2 eV and using the spectra of NFA with bandgap of 1.2 eV.

In order to predict the performance of single‐junction NFA OPVs based on active layers with different absorption spectra [by varying the optical band gap of the donor (*E*_opt,D_) and acceptor (*E*_opt,A_) materials within the BHJ], we generated absorption profiles of all active layers considered in this study using a linear combination of the extinction coefficient of the NFA and the donor materials, respectively (see example in **Figure** **3**). For this purpose, the *E*_opt,A_ and *E*_opt,D_ were shifted from 2.2 to 1.2 eV and from 2 to 1.2 eV, respectively. As illustrated in Figure 3b and Figure S5 (Supporting Information), shifting the donor and NFA bandgaps allows facile tuning of the onset of absorption of the active layer from which the value for *E*_opt,lowest_ can be inferred.

To account for the *V*_OC_ in our simulation, we used the LUMO energy of the NFA and the HOMO energy level of the donor to define the charge transfer state energy (*E*_CT_) using

(1) $$E_{\mathrm{CT}}={\mathrm{LUMO}}_{\mathrm{BHJ}}-{\mathrm{HOMO}}_{\mathrm{BHJ}}$$

In the case of NFA OPVs, it is often found that the energy of the CT state is close to that of the singlet exciton, which means that the excitons can separate efficiently upon negligible driving energies, i.e., *E*_opt,lowest_ − *E*_CT_ < 0.1 eV,^37^ where *E*_opt,lowest_ is the optical bandgap of the active layer as inferred from the absorption onset. In our simulation, the energy loss due to charge transfer is set to be 0.05 eV (Δ*E*_CS_), which is supported by previous studies of NFA OPVs.^14^, ^37^ From these considerations, the *E*_CT_ of the OPV devices is given by

(2) $$E_{\mathrm{CT}}=E_{\mathrm{opt},\mathrm{lowest}}-0.05\;\mathrm{eV}$$

Combining Equations (1) and (2) yields a relation for HOMO_BHJ_

(3) $${\mathrm{HOMO}}_{\mathrm{BHJ}}={\mathrm{LUMO}}_{\mathrm{BHJ}}-E_{\mathrm{opt},\mathrm{lowest}}+0.05\;\mathrm{eV}$$

Equation (3) above ensures that there is a driving force of 0.05 eV for the charge transfer step and that *E*_CT_ is as high as possible, without allowing energy transfer to the component with lowest energy. Furthermore, to assure that the formation of type‐II heterojunction is established between the donor and the NFA, we set the following two conditions: i) In the case of *E*_opt,D_ ≥ *E*_opt,A_, HOMO offset between donor and NFA is kept to 0.05 eV; ii) in the case of *E*_opt,D_ ≤ *E*_opt,A_, LUMO offset between donor and NFA is kept to 0.05 eV. Figure S6 (Supporting Information) provides further details on how the HOMO level of the donor and the LUMO level of the NFA are defined in this work.

In most of our single‐junction OPV simulations, the recombination rate constant *k* was set to 10^−12^ cm^3^ s^−1^—except for the calculations shown in Figure 6—since this is similar to values reported for the state‐of‐the‐art BHJ OPVs.^16^, ^17^, ^21^, ^24^, ^38^ The effective density of states of the active layer, *N*_0_, and the relative dielectric constant of the active layer, ε_r_, on the other hand, were set to 10^22^ cm^−3^ and 3.3, respectively.^16^ Moreover, we set the IQE value for the active layer to 0.95, assuming that 95% of absorbed photons generate free carriers that contribute to the photocurrent. This assumption is based on several recent reports on high‐efficiency NFA‐based OPVs for which IQE values in the range of 90–100% have been reported.^22^, ^39^ A summary of the input parameters used in our simulations is given in Table S2 (Supporting Information).

Having successfully generated the extinction coefficient of the BHJ layer across the entire visible–NIR spectrum, we studied the correlation between *E*_opt,D_ and *E*_opt,A_, and the optimum performance of single‐junction NFA OPVs. For all devices we assume that the work functions of the electrodes/interlayers are well aligned with the corresponding energy levels of the BHJ layer (Figure 1a) and form Ohmic contacts. For the charge transport we assumed balanced electron and hole mobilities (μ_h_ = μ_e_) of 5 × 10^−4^ cm^2^ V^−1^ s^−1^. The latter assumption and values are in line with recently reported data for the state‐of‐the‐art NFA‐based OPVs (see **Table** **2**).^22^, ^40^

**Table 2:** OPV performance metrics for representative high‐performance polymer donor:NFA photoactive blends. The charge mobility values were obtained from SCLC measurements

BHJ system	*FF* (%)	PCE (%)	Hole mobility [cm^2^ V^−1^ s^−1^]	Electron mobility [cm^2^ V^−1^ s^−1^]	Thickness [nm]	EQE [%]
PBDB‐TF:IT‐4F^4^	80.8	14.6	2.1 × 10^−4^	1.8 × 10^−4^	110	79–82
PDTB‐EF‐T:IT‐4F^2^	76.0	14.2	3.5 × 10^−4^	4.0 × 10^−4^	100	75–82
PBDB‐TF:IT:4F^9^	77.0	13.7	5.9 × 10−4a)	–	90	70–78
PM6:IT‐4F^39^	72.5	13.5	9.8 × 10^−4^	7.2 × 10^−4^	100	80–84
PBDB‐T‐SF:IT‐4F^22^	71.9	13.1	3.3 × 10^−4^	4.3 × 10^−4^	100	79–83
PBDB‐TF:IDTN^40^	78.0	12.2	5.1 × 10^−4^	5.7 × 10^−4^	100	60–70
PBDB‐T:NCBDT^41^	71.0	12.1	3.9 × 10^−4^	1.6 × 10^−4^	100	70–74
J61:m‐ITIC^42^	70.6	11.8	1.5 × 10^−4^	1.3 × 10^−4^	120	70–80
PvBDTTAZ:O‐IDTBR^43^	63.6	11.6	7.6 × 10^−4^	6.3 × 10^−4^	90	65–70
PBDB‐T:IT‐M^44^	70.0	11.5	3.3 × 10^−4^	1.1 × 10^−4^	100	60–80
FTAZ:INIC3^45^	67.4	11.5	2.0 × 10^−4^	1.4 × 10^−4^	100	72–77
PBDB‐T:ITCC^46^	71.0	11.4	2.1 × 10^−4^	6.7 × 10^−4^	110	75–78
PBDBT:ITIC^8^	74.2	11.2	2.1 × 10^−4^	3.1 × 10^−4^	100	74–76

^a)^ charge mobility values (dominated by faster carrier) was obtained from photo‐CELIV measurements.

**Figure 4:**
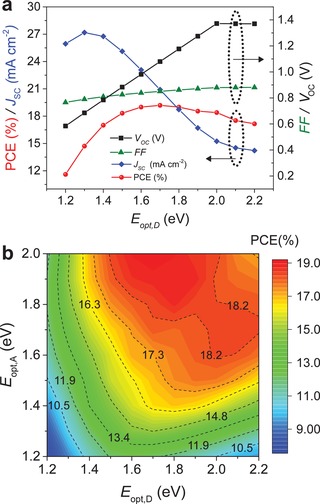
a) Evolution of the figures of merit of a single‐junction OPV device as a function of donor optical bandgap (*E*_opt,D_) with NFA optical bandgap (*E*_opt,A_) of 2.0 eV. This figure highlights the importance of maintaining balance between photocurrent gain and *V*_OC_ reduction. b) Efficiency prediction (plotted in color scale, with numbers on the contour lines representing PCE in %) for NFA OPVs as a function of the donor and NFA bandgap (*E*_opt,D_ and *E*_opt,A_). Input parameters: active layer thickness = 100 nm, and μ_h_ = μ_e_ = 5 × 10^−4^ cm^2^ V^−1^ s^−1^.

Using the aforementioned μ_h_, μ_e_, *k*, IQE, optical constant, and layer thickness values as the input parameters, our simulations predict that single‐junction NFA OPV cells may yield PCE values in excess of 18% (**Figure** **4**a). This prediction is particularly true for combinations of a donor (or NFA) and an NFA (or donor) with bandgaps of ≈1.7 and ≈2 eV, respectively (Figure 4b and Figure S7, Supporting Information). These results also highlight the importance for broad absorption characteristics by the BHJ layer in order to maximise *J*_SC_. To this end, broad absorption can, in principle, be facilitated by combining a wide bandgap donor with lower bandgap NFA material or vice versa.^2^, ^4^, ^21^, ^22^ However, one must always take into consideration the importance of maintaining optimum balance between the *J*_SC_ gain, due to enhanced absorption, and *V*_OC_ reduction owned to the use of narrower bandgap materials. For example, close analysis of Figure 4a reveals that for a donor bandgap of <1.4 eV, the *J_SC_* gains are not sufficient to outweigh the concurrent reduction in *V*_OC_ resulting to an overall reduction in PCE. Interestingly, even when the absorption of the donor and NFA materials overlap (Figure 4b), the cell's PCE remain relatively high and >10%. A further noticeable observation in Figure 4b is that the predicted PCE for OPVs based on wide bandgap (*E*_opt_ > 1.8 eV) donor and NFA materials can exceed 17% due to the characteristically high *V*_OC_ (>1.3 V) (Figure S7, Supporting Information), a value that has yet to be achieved/reported.

**Figure 5:**
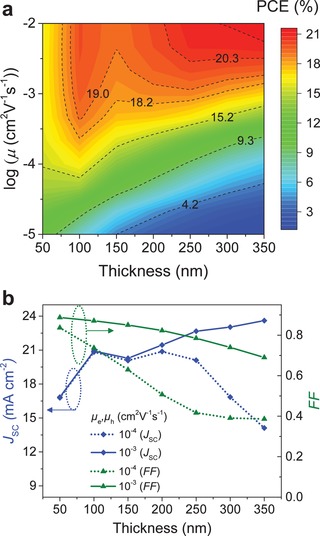
a) Efficiency prediction (plotted in color scale, with numbers on the contour lines representing PCE in %) for NFA OPVs as a function of charge mobility (μ = μ_h_ = μ_e_) and active layer thickness. b) Evolution of the *J*_SC_ and *FF* of a single‐junction OPV device as a function of active layer thickness for charge mobility of 10^−4^ and 10^−3^ cm^2^V^−1^ s^−1^.

Next, we examine how the hole and electron mobilities of the BHJ layer affect the single‐junction OPV cell performance. For this simulation, we set the active layer bandgap at the optimal value of 1.7 eV for the donor and 2 eV for the NFA while assuming equal hole and electron mobilities (μ_h_ = μ_e_) for both material components. In the case of unbalanced charge transport (μ_h_ ≠ μ_e_), the cell performance is found to be limited by the slower carrier with the role of the fastest one becoming irrelevant. **Figure** **5**a displays the predicted PCE for NFA OPVs as a function of charge carrier mobility (μ = μ_h_ = μ_e_) and active layer thickness (see also Figure S8 (Supporting Information) for other important figures of merit). Notably, PCE values of over 20% can be achieved in cells with layer thickness of >200 nm and μ > 10^−3^ cm^2^ V^−1^ s^−1^. Our simulations also reveal that for an OPV cell with 200 nm‐thick BHJ layer, achieving an *FF* ≥ 80% requires μ of ≥10^−3^ cm^2^ V^−1^ s^−1^ (Figure S8c, Supporting Information). Interestingly, however, even when mobility is reduced to 10^−4^ cm^2^ V^−1^ s^−1^, the cell's PCE remains high (≈18%) as long *k* is low (≈10^−12^ cm^3^ s^−1^) and IQE ≥ 95%. However, for practical applications, the cell's performance should exhibit reasonable tolerance to layer thickness variation, especially when large‐area manufacturing methods are employed, e.g., roll‐to‐roll printing.^4^, ^13^, ^40^ It can thus be inferred from the simulations that an excellent tolerance to layer thickness variation can easily be established for μ ≥ 10^−3^ cm^2^ V^−1^ s^−1^. For instance, close analysis of Figure 5a reveals that for μ = 10^−3^ cm^2^ V^−1^ s^−1^, PCE values of >19% can be achieved in cells with layer thickness of 100 nm. The PCE only modestly decrease to ≈18% when thicknesses are between 150–300 nm. On the other hand, for lower mobility values both *FF* and *J*_SC_ reduce with increasing layer thickness (Figure 5b). Specifically, for μ = 10^−4^ cm^2^ V^−1^ s^−1^, the cell's PCE drops from ≈16% to 7% with increasing of the layer thickness from 100 nm to 300 nm. To this end we note that although several NFA‐based OPVs with relatively high PCE (>12%) and *FF* (>0.75) have already been reported (Table 2), many of these cells are optimized for active layer thicknesses in the range of 100–120 nm and little or no work has been done on cells with significantly thicker BHJ layers.

Designing materials with planar conjugated backbones and high crystallinity is a common strategy for improving the charge carrier transport in organic semiconductors. Development of high mobility solution‐processed BHJ layers for OPVs, however, is a challenging task since both donor and NFA materials must form a bi‐continuous and interpenetrating network with reasonably small domain sizes (≈20 nm). This morphological feature is essential for efficient carrier photogeneration in organic semiconductors due to the short exciton diffusion lengths. On the other hand, planar molecules with extended conjugation are prone to phase separation, which in turn reduce the donor/NFA interface area required for efficient exciton dissociation.^13^, ^47^ Thus, in order to achieve the optimal BHJ layer morphology, the high carrier mobilities are often sacrificed by reducing the coplanarity of the conjugated molecules. As a result, the mobility of holes and electrons in the vast majority of state‐of‐the‐art BHJ OPVs are typically low and on the order of 10^−4^ cm^2^ V^−1^ s^−1^ (Table 2). Therefore, a critical challenge for current NFA‐based OPVs is to develop donor and NFA materials with balanced hole and electron mobility that exceeds 10^−3^ cm^2^ V^−1^ s^−1^ while avoiding strong phase separation upon blending.

**Figure 6:**
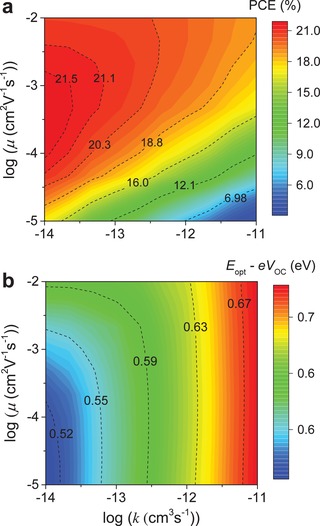
Influence of active layer mobilities (μ = μ_h_ = μ_e_) and recombination rate constant (*k*) on the a) efficiency (PCE) of single‐junction NFA OPV device and b) energy losses, *E*_opt_ − *V*_OC_ (*E*_opt,D_ = 1.7 eV, *E*_opt,A_ = 2.0 eV, Δ*E*_CS_ = 0.05 eV, thickness = 100 nm).

Apart from μ, *k* is also known to significantly affect the performance of BHJ OPVs.^16^, ^33^ Thus, understanding the concurrent influence of carrier mobility and recombination on the cell's performance, is essential. As illustrated in **Figure** **6**a, the general trend is that decreasing *k* improves the cell's PCE. For instance, in a BHJ layer with balanced hole and electron mobility of 5 × 10^−4^ cm^2^ V^−1^ s^−1^, reducing *k* from 10^−11^ to 10^−13^ cm^3^ s^−1^ results in a significant increase in the cell's PCE from ≈16.8% to ≈20.9%. A further increase of the PCE to ≈21.8% can be achieved by reducing *k* further to 10^−14^ cm^3^ s^−1^. However, our simulations also show that single‐junction OPV cells incorporating BHJs with the highest μ and the lowest *k* may not always yield optimum PCEs (Figure 6a,b). This is somewhat counterintuitive since it is generally assumed that simultaneously reducing *k* and increasing μ in a BHJ OPVs will always result to performance improvements. However, close examination of Figure 6a,b reveals that when *k* < 10^−13^ cm^3^ s^−1^, raising μ to >10^−3^ cm^2^ V^−1^ s^−1^ results to simultaneous reduction in *V*_OC_ and as such PCE. We note however, that in our simulations *k* is assumed to be independent of μ because we do not employ the Langevin recombination model.^16^

Since our model does not account for geminate recombination and electric field‐dependent charge separation effects, the *V*_OC_ drop observed at higher μ most likely stems from surface recombination (see discussion related to Figure S9 in the Supporting Information).^48^, ^49^, ^50^ This adverse effect can in principle be overcome through the use of selective contacts.^27^ To this end, the use of optimized ETL and HTL can help improve the OPV efficiency by selectively transporting electrons and holes, respectively, from the active layer to the corresponding electrodes, whilst simultaneously preventing recombination of minority carriers at the contacts. Indeed, when selective contacts are employed, the highest μ and the lowest *k* yield optimum PCE values (Figure S9d, Supporting Information). However, we note that in the case of non‐negligible bulk recombination (i.e., *k* = 10^−12^ cm^3^ s^−1^), the predicted PCE values for OPVs employing selective contacts is not significantly different from those predicted for cells with metallic contacts (Figure S10, Supporting Information).

To this end, the range of experimentally measured *k* values for fullerene‐based BHJ solar cells is between 10^−13^ to 10^−10^ cm^3^s^−1^ 16 with the nongeminate recombination rate being determined, primarily, by the area of the donor/NFA heterointerface.^51^ This conclusion is in accordance with a recent study of the recombination processes in ternary polymer/polymer or polymer/fullerene BHJs where more phase‐separated blends were shown to exhibit lower nongeminate *k* values.^52^, ^53^ However, approaches to reducing *k* are not clearly laid out at present. Thus, development of experimental methods that can provide accurate and reliable characterization of the nongeminate recombination kinetics are needed in order to establish meaningful structure‐property relationships. Recently, Heiber et al. developed an impedance‐photocurrent device analysis (IPDA) technique to measure charge transport and recombination with OPV devices under operation with high accuracy and reliability.^54^ Such experimental techniques might soon allow researchers to identify the necessary design rules to reduce recombination rate constant in state‐of‐the‐art organic BHJ systems and OPV devices.

**Figure 7:**
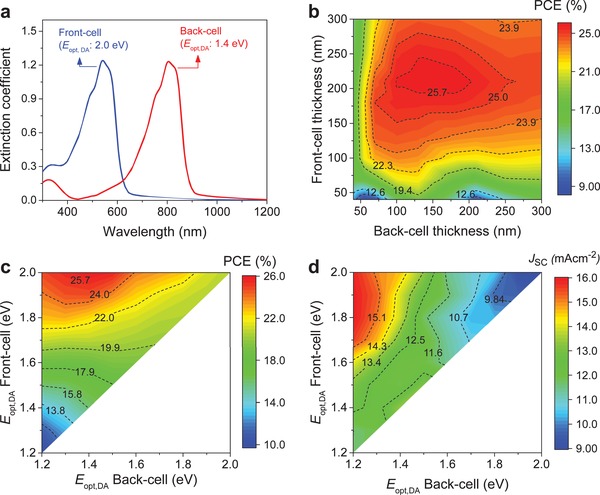
a) Absorption profiles considered for two‐terminal tandem OPV calculations with the front‐ and back‐cell optical bandgaps (*E*_opt,DA_) of 2 and 1.4 eV, respectively (donor and NFA absorptions in both cells were considered to overlap). b) Efficiency prediction of two‐terminal NFA‐based tandem OPVs (the optical bandgaps for the front‐ and back‐cell were 2 and 1.4 eV, respectively) simulated from optical and electrical modelling. Evolution of the c) PCE and d) *J*_SC_ of two‐terminal NF‐based tandem OPV cells as a function of front‐cell and back‐cell optical bandgaps (*E*_opt,DA_) assuming *k* = 10^−12^ cm^3^ s^−1^, and μ_n_ = μ_p_ = 10^−3^ cm^2^ V^−1^ s^−1^.

The PCE of OPVs can be improved further using multijunction architectures where multiple subcells with complementary optical absorption spectra are stacked on top of each other in order to harness photons from a wider range of the solar spectrum. Indeed, multijunction OPV devices have been investigated widely and certified PCE values of ≈17.29% have been reported for 2T tandem cells.^3^ The latter is significantly higher than the record PCE values reported to date for single‐junction OPVs.^4^ In an effort to better understand the practical efficiency limits of tandem OPVs, we combined optical transfer matrix modelling with the 1D *J–V* simulations of single‐junction NFA‐based OPV cells.^55^ First, the *J–V* curve of each single‐junction device (for different layer thicknesses) were simulated using the numerical simulator employing the previously discussed parameters (i.e., μ_n_ = μ_p_ = 10^−3^ cm^2^ V^−1^ s^−1^, *k* = 10^−12^ cm^3^ s^−1^, with IQE set to 95%). As shown in **Figure** **7**a, the absorption profiles of the donor**/**NFA BHJ systems used for the front and back‐cells, were set to overlap by selecting their optical bandgaps as 2 and 1.4 eV, respectively. Subsequently, transfer matrix modelling and Kirchhoff's law were applied to construct the overall *J–V* curve for the multijunction OPV device.^55^, ^56^, ^57^, ^58^ Figure 7b shows the relation between the subcells' thicknesses and predicted PCE. Figure 7c reveals that the PCE for tandem OPVs is maximized when the optical bandgap of the front and back‐cell is in the range of 1.8–2 eV and 1.2–1.5 eV, respectively. The result shown in Figure 7c suggests that development of highly efficient front‐cell with wide optical bandgap (1.8–2 eV) is critically needed as most of the current developments are using front‐cell with optical bandgap <1.7 eV.^3^, ^5^, ^6^, ^7^ As shown in Figure 7b,c and Table S2 (Supporting Information), our simulations indicate that maximum PCE values as high as 25% can be expected for optimized two‐terminal tandem OPV cells (i.e., by combining optical bandgap of front and back‐cell of 2 and 1.4 eV, respectively) with μ_n_ = μ_p_ = 10^−3^ cm^2^ V^−1^ s^−1^, and *k* = 10^−12^ cm^3^ s^−1^. We also note (Table S2, Supporting Information) that even in the case of nonoverlapping absorption characteristics (Figure S12, Supporting Information), the maximum PCE remain almost identical to the overlapping subcell values (Figure 7a). Finally, Figure 7d shows that the *J*_SC_ in NFA‐based two‐terminal tandem OPVs can easily exceed 15 mA cm^−2^ if the bandgaps of the front and back‐cells are carefully chosen/engineered. For comparison, the *J*_SC_ of state‐of‐the‐art two‐terminal tandem OPV reported recently is ≈14.3 mA cm^−2^.^3^ Therefore, it becomes clear that for the development of NFA‐based tandem OPVs with PCE > 20% active materials with higher carrier mobility and carefully tuned absorption characteristics are required. Our analysis also suggests that emerging OPV technologies may ultimately rival, in terms of PCE, traditional inorganic PV technologies.

## 3 Conclusion

In summary, with the aid of 1D device simulations, we examined the impact of important material parameters on the operating characteristics of NFA‐based OPV cells. Studied parameters included: i) the electron and hole mobilities, ii) the active layer thickness, and iii) the recombination rate constant. We found that with the already reported hole and electron mobilities of 5 × 10^−4^ cm^2^ V^−1^ s^−1^, single‐junction NFA‐based OPVs can potentially reach PCEs of over 18% if nongeminate recombination rate constant could be reduced to 1 × 10^−12^ cm^3^ s^−1^ and IQE increased to 95%. Moreover, PCE values in excess of 20% is shown to be within reach even for single‐junction OPVs either by increasing the charge carrier mobility and active layer thickness to >10^−3^ cm^2^ V^−1^ s^−1^ and to >200 nm, respectively, or by reducing the recombination rate constant (*k*) to ≤1 × 10^−13^ cm^3^ s^−1^. Finally, by combining optical transfer matrix modelling with the aforementioned singe‐cell *J–V* simulations, we were able to model two‐terminal tandem NFA‐based OPV cells. Our simulations predict that carefully engineered tandem cells may achieve PCE values of over 25% if the electron (μ_e_) and hole (μ_h_) mobilities in the donor and acceptor materials used for the individual subcells are balanced (μ_e_ = μ_h_) and ≥10^−3^ cm^2^ V^−1^ s^−1^, while *k* remains low and around 10^−12^ cm^3^ s^−1^. The present study provides the first comprehensive description of the relationship between fundamental material properties and device design aspects while highlighting the key design rules for the development of single and multijunction NFA‐based OPVs with optimal performance characteristics.

## 4 Experimental Section

*Solar Cell Fabrication (PBDB‐T:ITIC and PBDB‐T‐SF:IT‐4F)*: The materials were purchased from Solarmer Materials Inc. Indium tin oxide (ITO) coated glass substrates (Kintec Company, 10 Ω sq.^−1^) were cleaned by sequential ultrasonication in dilute Extran 300 detergent solution, deionized water, acetone, and isopropyl alcohol for 20 min each. These substrates were then cleaned by UV‐ozone treatment for 30 min. Then, a thin layer (≈30 nm) of poly(2,3‐dihydrothieno‐1,4‐dioxin)‐poly(styrenesulfonate) (PEDOT:PSS) was spin‐cast onto the UV‐treated substrates, dried on a heating plate at 120 °C for 15 min, and the substrates were then transferred into a dry nitrogen glove box (<10 ppm O_2_). Active layer (ratio 1:1, 14 mg mL^−1^ in chlorobenzene) were then spun at 2000 rpm for 30 s (active‐layer thickness 90–100 nm). A layer of 4 nm of Phen‐NaDPO^59^ as ETL was spun from isopropanol solution (0.5 mg mL^−1^) on top of the active layer. Finally, the samples were placed in a thermal evaporator and 100 nm of aluminum were then thermally evaporated at 5 × 10^−6^ mbar.

*Device Characterization: J–V* measurements of solar cells were performed in a N_2_ filled glove box using a Keithley 2400 source meter and an Oriel Sol3A Class AAA solar simulator calibrated to 1 sun, AM1.5G, with a KG‐5 silicon reference cell certified by Newport. The carrier mobilities (hole and electron mobilities) of PBDB‐T:ITIC and PBDB‐T‐SF:IT‐4F BHJ devices were determined by fitting the dark currents of hole/electron‐only diodes to the SCLC model. Hole‐only diode configuration: Glass/ITO/PEDOT/BHJ/MoO_3_/Ag. Electron‐only diode configuration: Glass/ITO/*a*‐ZnO/BHJ/Phen‐NaDPO/Al. External quantum efficiency (EQE) was characterized using a specially designed EQE system (PV measurement Inc.). Measurements were performed at zero bias by illuminating the device with monochromatic light supplied from a Xenon arc lamp in combination with a dual‐grating monochromator. The number of photons incident on the sample was calculated for each wavelength by using a silicon photodiode calibrated by NIST (The National Institute of Standards and Technology). The IQE spectra were calculated from EQE spectra using the relation: IQE = EQE/(1 − Reflectance − Parasitic Absorption). The reflectance spectra were collected with the integrating sphere using the same EQE system while the parasitic absorption spectra were obtained from transfer matrix modelling.

*TDCF Measurement*: The home‐built TDCF setup uses the second harmonic (532 nm) of an actively Q‐switched sub‐ns Nd:YVO4 laser (INNOLAS piccolo AOT) operating at a repetition rate of 5 kHz as excitation source. To minimize the RC response time, a small device area of 1 mm^2^ is used in TDCF experiments. The samples were measured under dynamic vacuum conditions to avoid any degradation. A Keysight S1160A function generator was used to provide the prebias and extraction bias pulses, while a Keysight four channel digital oscilloscope (Infiniivision MSOX3034T) was used to measure the current response of the device.

*Numerical Device Simulator*: 1D numerical drift‐diffusion device simulator (Setfos 4.4 from FLUXiM AG) was used to predict the single‐junction OPV device *J–V* curves. The optical constants (refractive index and extinction coefficient) for the active layers were collected by variable angle spectroscopic ellipsometry (VASE) with an M‐2000 ellipsometer (J.A. Woolam Co., Inc). The active layers were cast on clean silicon substrates coated with SiO_2_ (100 nm). The VASE measurements were performed with incident angles being varied from 50° to 80° in steps of 5° relative to the samples. The software Complete Ease (J.A. Woolam Co., Inc) was used to process all collected data, and the optical constants were inferred from the B‐splines model.

*Modelling of Multijunction Device J–V Curves*: In all tandem OPV device simulations, input characteristics obtained from optical transfer matrix modeling (subcell *J*_SC_ values) were combined with the analysis of the *J–V* curves for single‐junction devices simulated by the 1D numerical drift‐diffusion device simulator (Setfos 4.4 from FLUXiM AG). The *J–V* curves of the multijunction OPV devices were produced using Kirchhoff's laws, and the figures of merit were subsequently represented in 3D maps depicting performance as a function of subcell thicknesses and optical gap of the front‐ and back‐cells.

## Conflict of Interest

The authors declare no conflict of interest.

## Supporting information

Supplementary
