# Supplementary material for: Key Parameters Requirements for Non‐Fullerene‐Based Organic Solar Cells with Power Conversion Efficiency >20%
Source: Adv Sci (Weinh). 2019 Mar 10;6(9):1802028. doi: 10.1002/advs.201802028 (PMC6498106; doi:10.1002/advs.201802028)
Supplement: Supplementary file 1 — Supplementary [file ADVS-6-1802028-s001.pdf]

Copyright WILEY-VCH Verlag GmbH & Co. KGaA, 69469 Weinheim, Germany, 2019.

Supporting Information

for *Adv. Sci.,* DOI: 10.1002/advs.201802028

Key Parameters Requirements for Non-Fullerene-Based Organic Solar Cells with Power Conversion Efficiency >20%

Yuliar Firdaus, Vincent M. Le Corre, Jafar I. Khan, Zhipeng Kan, Frédéric Laquai, Pierre M. Beaujuge, and Thomas D. Anthopoulos**

Copyright WILEY-VCH Verlag GmbH & Co. KGaA, 69469 Weinheim, Germany, 2019.

Supporting Information

Key Parameters Requirements for Non-Fullerene Based Organic Solar Cells with Power Conversion Efficiency >20%

Yuliar Firdaus, Vincent M. Le Corre, Jafar I. Khan, Zhipeng Kan, Frédéric Laquai, Pierre M Beaujuge and Thomas D. Anthopoulos*

S1. Carrier Mobility Measurements

The carrier mobilities (hole and electron mobilities) of optimized BHJ films (PBDB-T:ITIC and PBDB-T-SF:IT-4F) were determined by fitting the dark currents of hole/electron-only diodes to the space-charge-limited current (SCLC) model. Hole-only diode configuration: Glass/ITO/PEDOT /BHJ/MoO_3/Ag. First, a thin layer (ca. 30 nm) of PEDOT:PSS was spin-coated at 4000 rpm for 30 s onto the ITO-coated glass in air and then baked at 150º C for 10 min. After deposition of the active layer on the PEDOT:PSS-coated substrates, MoO_3 (5 nm, 0.3 Å s^{-1}) and Ag (100 nm, 5 Å s^{-1}) were deposited via thermal evaporation in a vacuum chamber at a base pressure of 2×10^{-6} mbar. Electron-only diode configuration: Glass/ITO/a-ZnO/ /BHJ/phen-NADPO/Al. The active layers were deposited on top of the ZnO/ITO-coated substrates, and phen-NaDPO (2000 rpm from 0.5 mg/ml solution in isopropanol) and Al (100 nm, 5 Å s^{-1}) were subsequently deposited via thermal evaporation in a vacuum chamber at a base pressure of less than 2×10^{-6} mbar. Active-layer thicknesses were measured with a Tencor surface profilometer. The carrier mobilities were inferred from fitting the data to the Murgatroyd expression:

$$J\,(V) = \frac{9}{8}\varepsilon_0\varepsilon_r\mu_0 \exp\left(0.89\beta\sqrt{\frac{V - V_{bi}}{L}}\right)\frac{(V - V_{bi})^2}{L^3} \tag{S1}$$

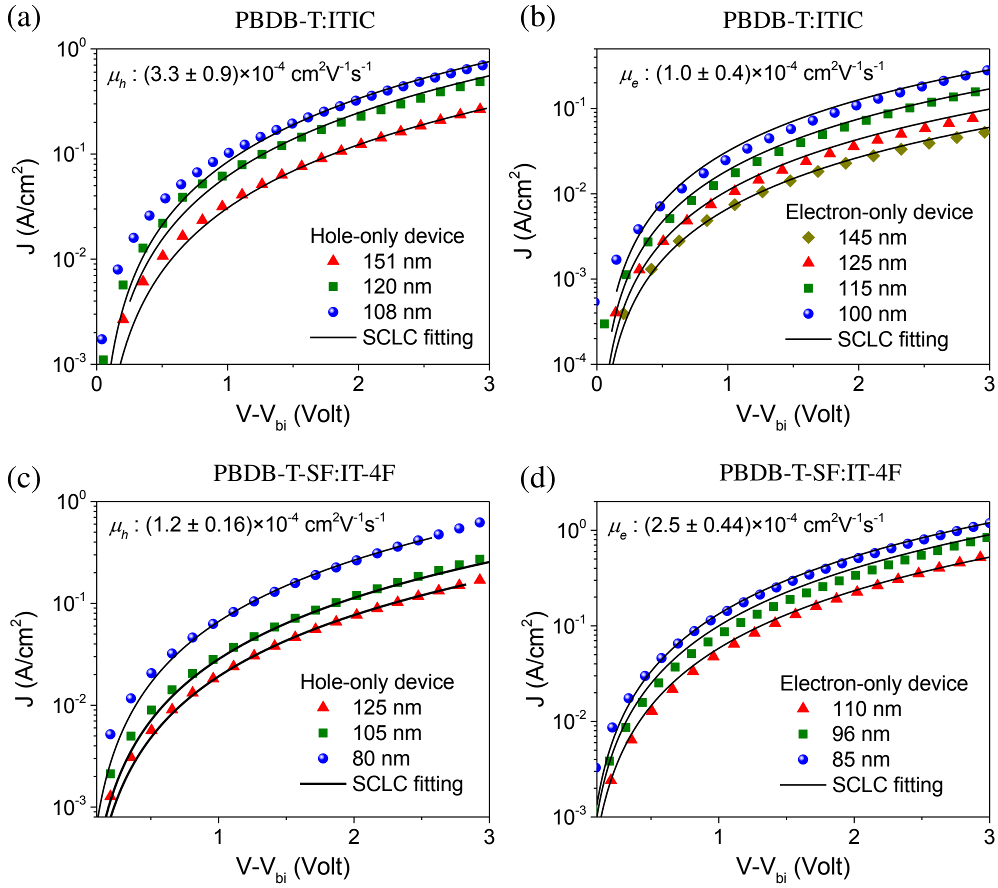

Figure S1. Dark current density-voltage (*J-V*) characteristics for (a) hole-only, and (b) electron-only diodes of PBDB-T:ITIC and PBDB-T-SF:IT-4F systems. The hole-only device structure is Glass/ITO/PEDOT:PSS/BHJ/MoO_3/Ag. The electron-only device structure is Glass/ITO/*a*-ZnO/BHJ/Phen-NaDPO/Al. The solid lines are fits to the experimental data according to the Murgatroyd expression.

S2. Simulator validation

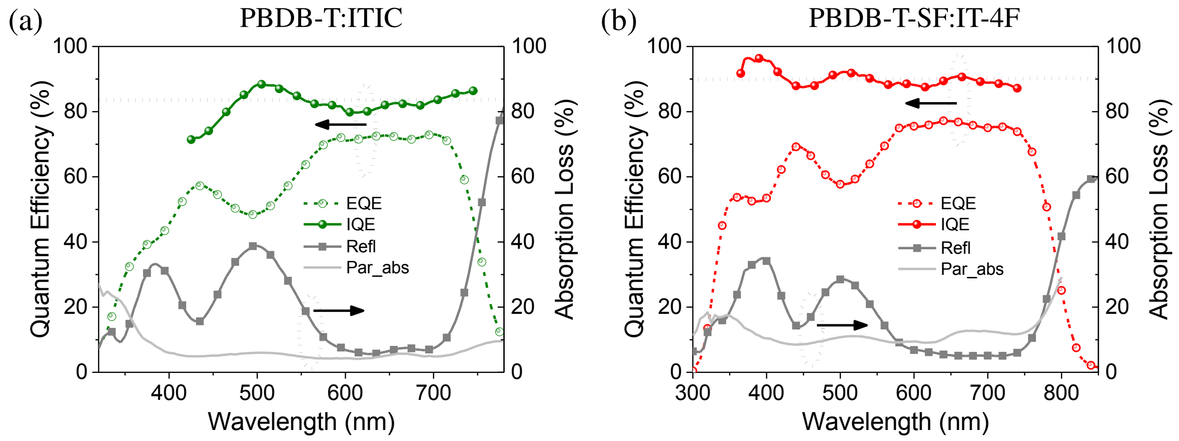

Figure S2. EQE, IQE, reflectance and parasitic absorption of BHJ solar cells of (a) PBDB-T:ITIC and (b) PBDB-T-SF:IT-4F devices.

Table S1. Input parameters (charge carrier mobility, recombination rate constant, and IQE) used for the *J-V* curve simulation of PBDT(T)[2F]T:ITIC, PDBD-T:ITIC, and PBDB-T-SF:IT-4F shown in Figure 2. The recombination rate constant obtained from time-delayed collection field (TDCF) measurement (k_{TDCF}) also shown.

Donor:Acceptor (**D:A**)	μ_h	μ_e	k(sim)	IQE	k_{TDCF}
	$cm^2V^{-1}s^{-1}$	$cm^2V^{-1}s^{-1}$	cm^3s^{-1}	%	cm^3s^{-1}
PBDT(T)[2F]T:ITIC	3.0×10^{-5}	1.2×10^{-5}	1.2×10^{-12}	83	5.6×10^{-12}
PBDB-T:ITIC	4.0×10^{-4}	1.0×10^{-4}	6.8×10^{-12}	85	2.8×10^{-11}
PBDB-T-SF:IT-4F	1.2×10^{-4}	2.5×10^{-4}	3.4×10^{-12}	90	1.6×10^{-11}

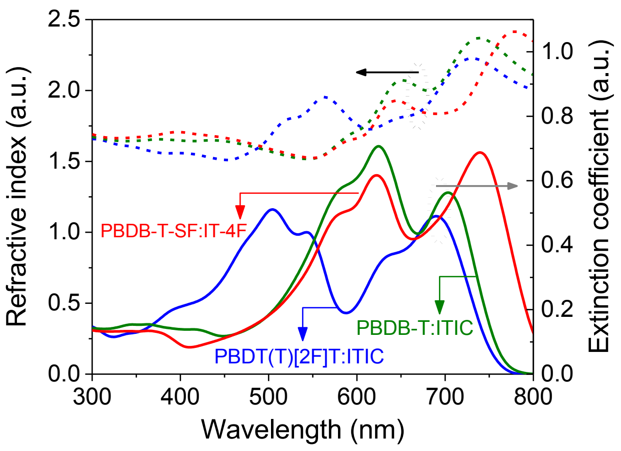

Figure S3. Optical constant profiles (extinction coefficient, and refractive index) obtained from PBDT(T)[2F]T:ITIC, PBDB-T:ITIC, and PBDB-T-SF:IT-4F films obtained from ellipsometry measurements.

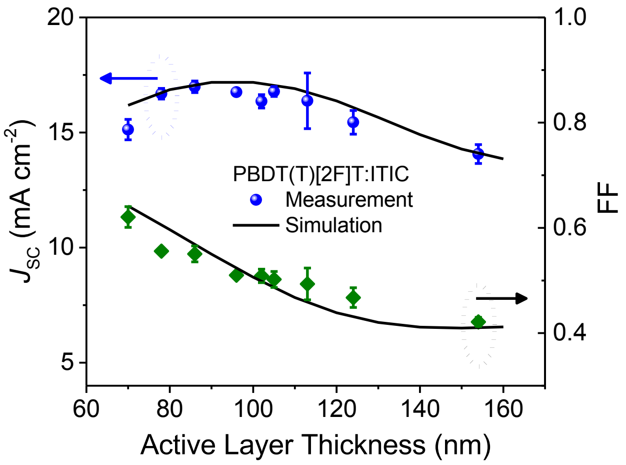

Figure S4. Experimentally measured (symbol) and simulated (solid line) current density (J_{SC}) and fill-factor (*FF*) versus active layer thickness PBDT(T)[2F]T:ITIC BHJ solar cell devices.

S3. Efficiency limit calculation (single-junction)

Table S2: Input drift-diffusion parameters for the numerical simulations.

Input parameters	BHJ	HTL	ETL
ε_r	3.3	10	10
N_0 (cm^{-3})	10^{22}	10^{22}	10^{22}
Thickness (nm)	100	10	10
IQE	0.95	/	/
μ_p (cm^2 V^{-1} s^{-1})	vary	10	1
μ_n (cm^2 V^{-1} s^{-1})	vary	1	10

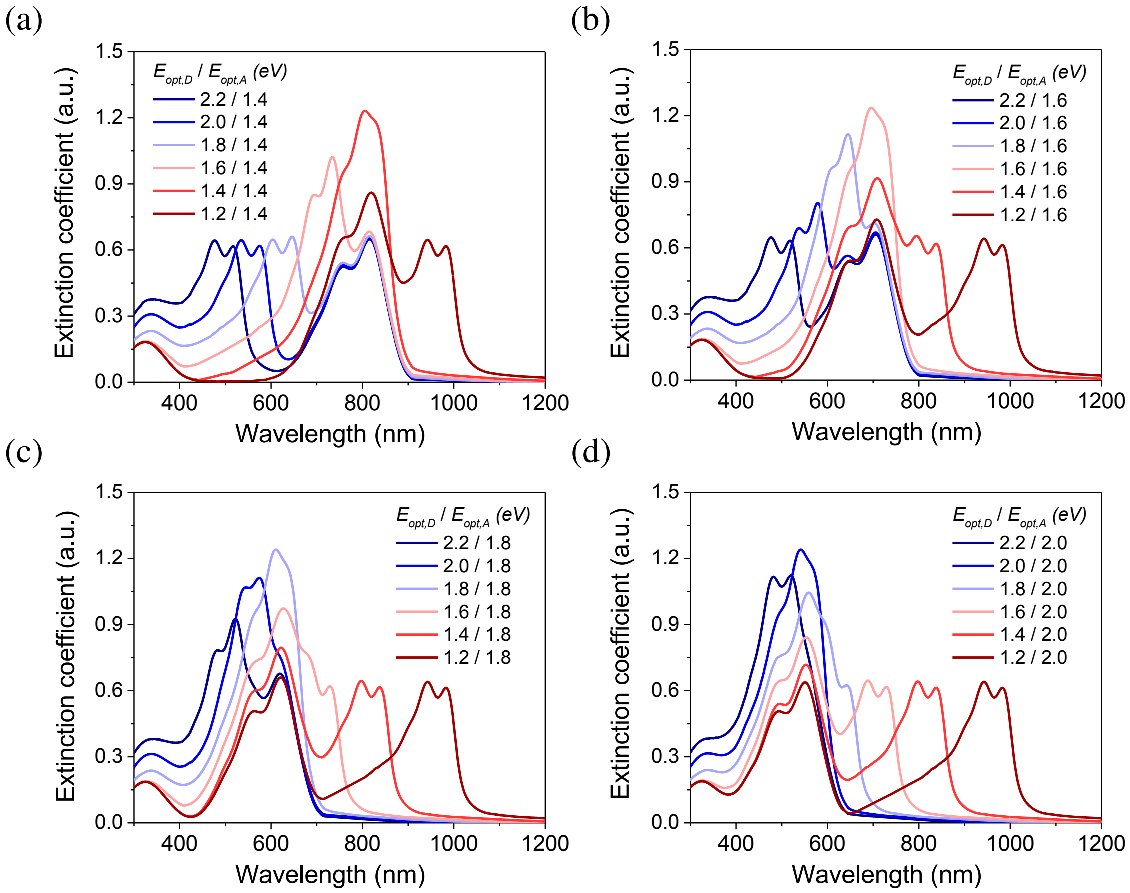

Figure S5. Extinction coefficient profiles of active-layer blends used in this study as a result of linear combination of the extinction coefficient of donor ($E_{opt,D}$ from 2.2 eV to 1.2 eV) and different NFA bandgap ($E_{opt,A}$) of : (a) 1.4 eV, (b) 1.6 eV, (c) 1.8 eV and (d) 2.0 eV.

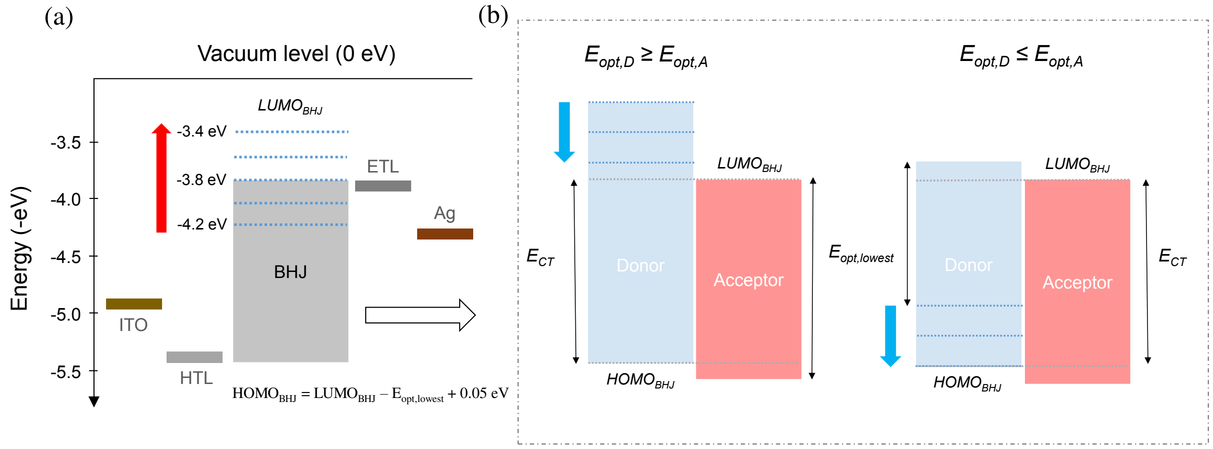

Figure S6. (a) Schematic energy diagram for the single-junction OPV device with the BHJ layer drawn as a single-phase material. As example, the LUMO level was set to -4.2 eV and -3.4 eV for acceptor material with bandgap of 1.2 eV and 2.0 eV, respectively. The HOMO level is given by:

$\mathrm{HOMO_{BHJ}} = \mathrm{LUMO_{BHJ}} - \mathrm{E_{opt,lowest}} + 0.05\ \mathrm{eV}$. (b) Further details on how the HOMO and LUMO level were set in the case of $E_{\mathrm{opt,D}} \geq E_{\mathrm{opt,A}}$ and $E_{\mathrm{opt,D}} \leq E_{\mathrm{opt,A}}$.

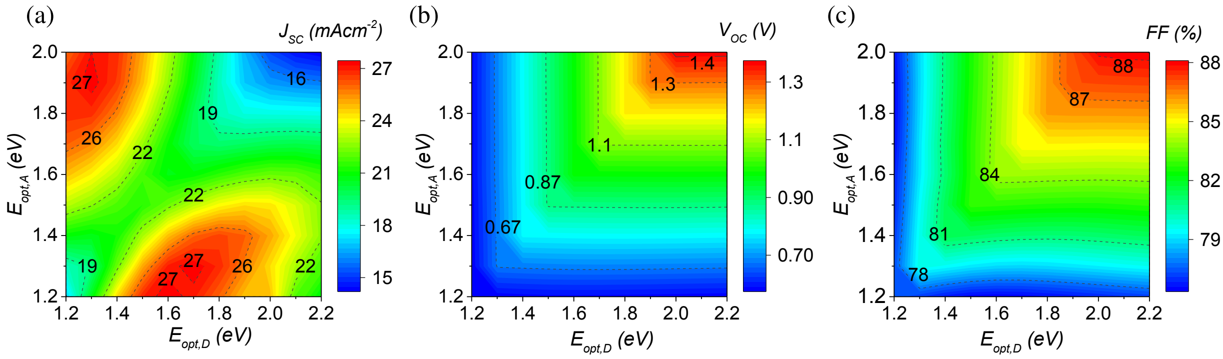

Figure S7. (a) J_{SC}, (b) V_{OC}, and (c) *FF* prediction (plotted in colour scale, with numbers on the contour lines representing J_{SC}, V_{OC}, *and FF*, respectively) for NFA OPVs as a function of the **D** and **A** bandgap. Input parameters: $\mu_h = \mu_e = 5\times10^{-4}$ cm^{2}V^{-1}s^{-1}.

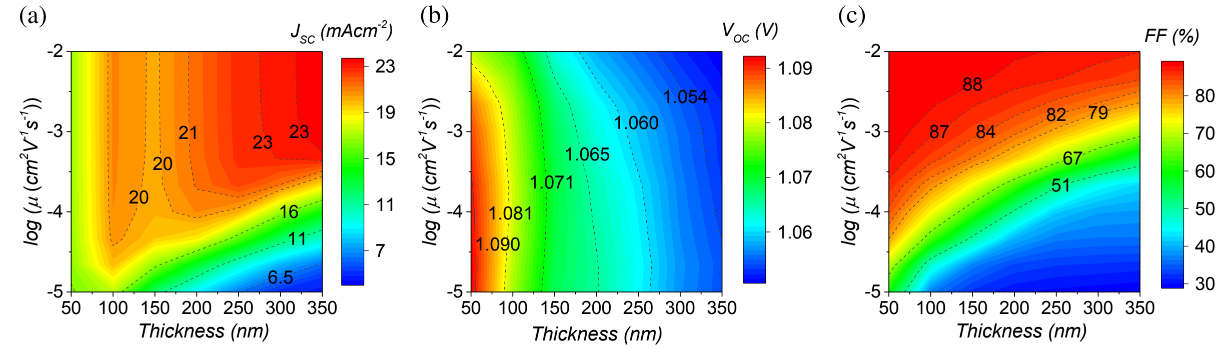

Figure S8. (a) J_{SC}, (b) V_{OC}, and (c) *FF* prediction (plotted in colour scale, with numbers on the contour lines representing J_{SC}, V_{OC}, *and FF*, respectively) for NFA OPVs as a function of the charge mobility ($\mu = \mu_h = \mu_e$) and active layer thickness.

S4. Influence of dark-current on V_{OC}

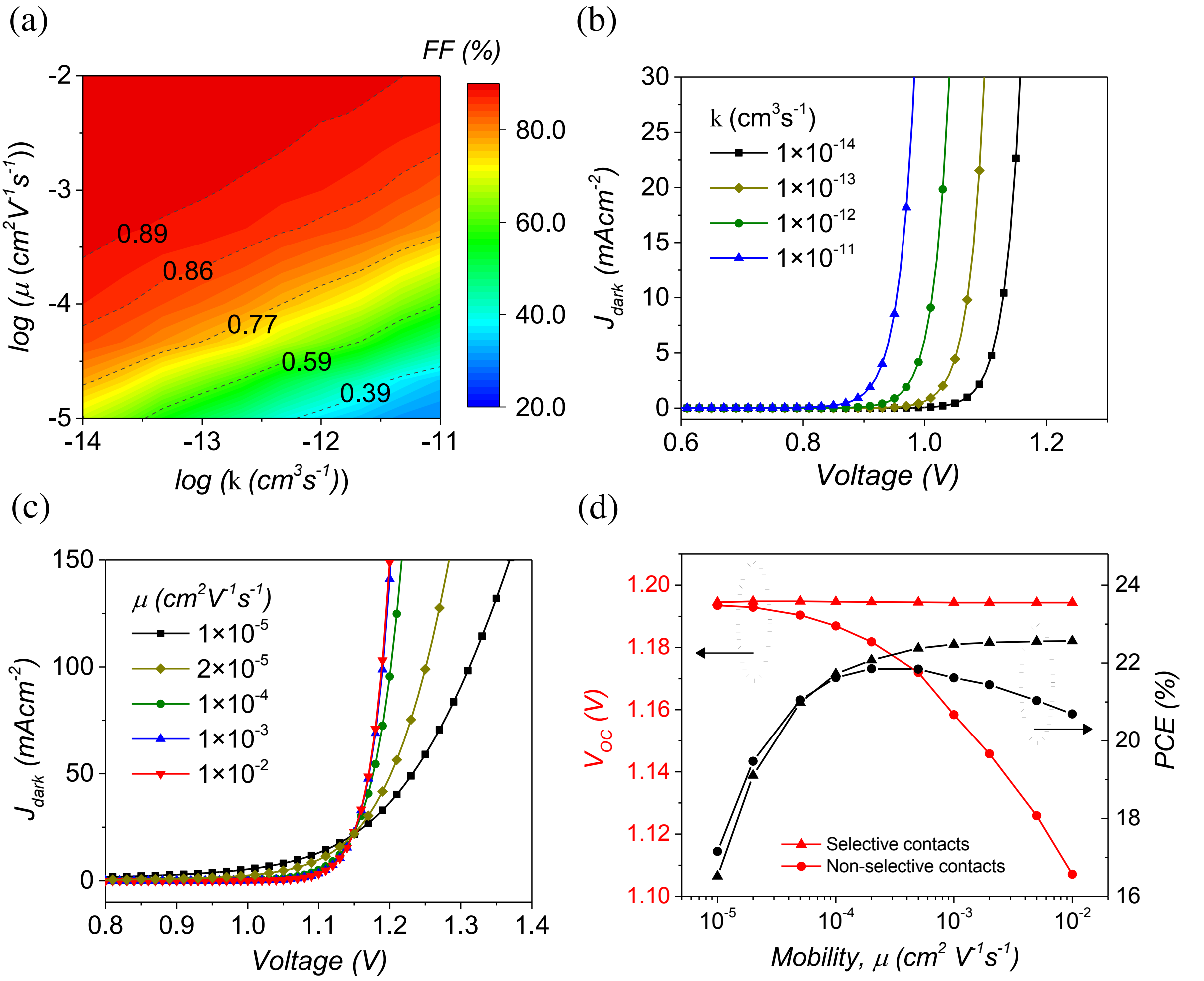

Figure S9. (a) Influence of active layer mobilities (μ= μ_h= μ_e) and recombination rate constant (*k*) on the (a) fill-factor (*FF*) of single-junction NFA OPV device. (b) Dependence of the dark current J_{dark} on the recombination rate constant (*k*) in single-junction OPV devices ($E_{opt,D}$= 1.7 eV, $E_{opt,A}$= 2.0 eV); here μ= μ_n= μ_p= 10^{-2} cm^2 V^{-1} s^{-1}. (c) Dependence of J_{dark} on the active layer mobilities in the absence of non-geminate recombination (*i.e. k*= 10^{-14} cm^3 s^{-1}). (d) Mobility dependent of V_{OC} and *PCE* in the case of selective contacts and metallic contacts (*k*= 10^{-14} cm^3 s^{-1}). For the case of selective contacts, both electron barrier height of the HTL and hole barrier height of the ETL are set to 0.6 eV.

Two different contributions influence dark current (J_{dark}) and, in turn, affect V_{OC}. A first contribution to J_{dark} is the bulk recombination current, which is mobility-independent but, as illustrated in Figure S9b, depends strongly on *k* – the proportionality constant linked to the non-geminate recombination rate (R = knp). The second contribution to J_{dark} is made apparent when *k* is set to 0 (*i.e.* no non-geminate recombination) and the device *J-V* curve is simulated in the dark; analysis described in

Figure S9c. In forward bias (with k = 10^{-14} cm^3s^{-1}), the contributor to J_{dark} is the injection of carriers from the device contacts and the transport of these carriers across the active layer to the opposite contact, where they recombine, which we call surface recombination. As shown in Figure S9c, the magnitude of the dark injection current connected to the charge-carriers diffusing across the active layer is proportional to the carrier mobility. When k = 0 or <10^{-13} cm^3 s^{-1}, the bulk recombination current is negligible and the dark injection current becomes the main contributor to the V_{OC} losses. In this case, raising the carrier mobilities of the active layer increases the magnitude of J_{dark} and decreases V_{OC}. In contrast, as illustrated in Figure 6b, if the bulk recombination current determines J_{dark}, increasing the carrier mobilities of the active layer has little to no effect on the V_{OC} because the recombination current is mobility-independent.

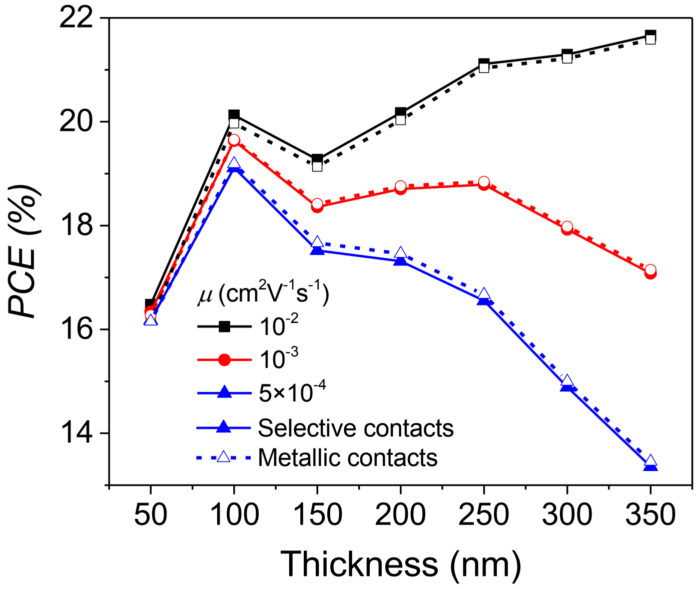

Figure S10. Efficiency prediction for NFA-based OPVs as a function of active layer thickness in the case of selective and metallic contacts where the bulk-recombination is not negligible (k= 10^{-12} cm^3 s^{-1}).

S5. Efficiency limit calculation (2-T tandem-cell)

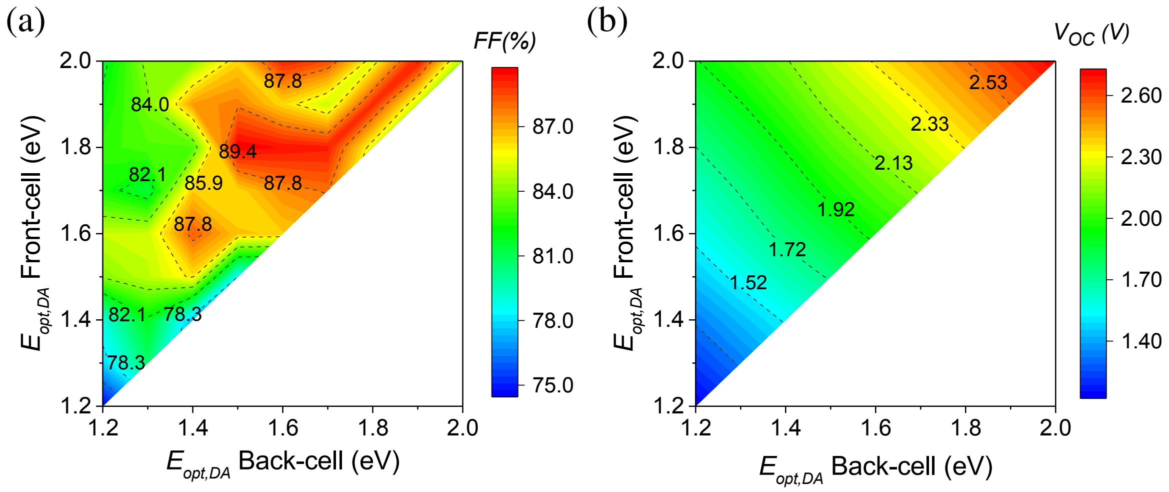

Figure S11. Evolution of the (a) *FF*, and (b) V_{OC} of 2-T NFA-tandem OPV devices as a function of front-cell and back-cell optical bandgaps ($E_{opt,DA}$); $k = 10^{-12}$ cm^3 s^{-1}, case for $\mu_n = \mu_p = 10^{-3}$ cm^2 V^{-1} s^{-1}.

Table S2. Simulated 2-T NF-tandem OPV performance for different absorption profiles (overlap vs non-overlap donor/NFA absorption spectra). *Optimal thickness of the sub-cells.

Front-cell			Back-cell			J_{SC}	V_{OC}	FF	PCE
$E_{opt,D}$ (eV)	$E_{opt,A}$ (eV)	thick (nm)*	$E_{opt,D}$ (eV)	$E_{opt,A}$ (eV)	thick (nm)*	mAcm^{-2}	V	%	%
2.1	1.7	90	1.6	1.4	110	13.7	1.86	87.0	22.1
1.7	1.7	70	1.4	1.4	130	13.0	1.85	86.5	20.8
2.1	1.8	130	1.6	1.4	110	13.9	1.95	86.2	23.3
1.8	1.8	100	1.4	1.4	250	13.9	1.93	83.3	22.4
2.1	1.9	190	1.6	1.4	130	14.4	2.03	84.6	24.6

1.9	1.9	180	1.4	1.4	130	14.0	2.03	87.3	24.7
2.1	2.0	230	1.6	1.4	130	13.9	2.13	84.9	25.0
2.0	2.0	210	1.4	1.4	180	14.6	2.12	84.2	26.0

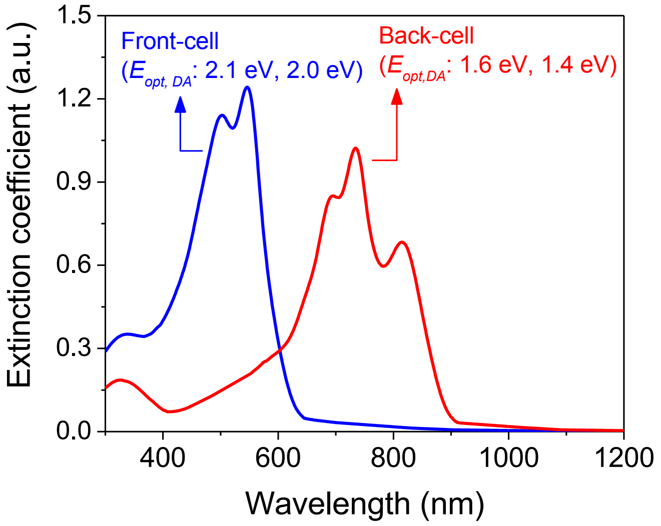

Figure S12. Absorption profiles considered for tandem OPV devices calculation for non-overlapping donor and NFA absorption (complementary absorption).
